# Cultural worldviews and waste sorting among urban Chinese dwellers: the mediating role of environmental risk perception

**DOI:** 10.3389/fpubh.2024.1344834

**Published:** 2024-04-05

**Authors:** Lin Cai, Qingjun Li, Erya Wan, Menglin Luo, Siwen Tao

**Affiliations:** ^1^School of Community for the Chinese Nation, Southwest Minzu University, Chengdu, China; ^2^School of Marxism, Sichuan Institute of Industrial Technology, Deyang, China; ^3^School of Global Health, Shanghai Jiao Tong University, Shanghai, China; ^4^School of Pharmaceutical, Southern Medical University, Guangzhou, China

**Keywords:** cultural worldview, waste sorting, environmental risk perception, urban dwellers, cultural theory

## Abstract

**Objective:**

Waste sorting has received considerable attention in recent decades. However, research on the mechanisms underlying the relationships among cultural worldview, environmental risk perception, and waste sorting is rather scarce. This study aims to explore the cultural worldviews, environmental risk perception, and waste sorting among urban Chinese and their mechanisms.

**Methods:**

This was a cross-sectional study involving 744 urban Chinese residents (371 men and 373 women). A questionnaire was utilized to measure cultural worldviews, environmental risk perception, and waste sorting. Pearson correlation analysis and structural equation modeling were used to examine the relationship between cultural worldviews, perceptions of environmental risk, and waste sorting.

**Results:**

Waste sorting had a relatively insignificant negative relationship with fatalism and individualism. The correlation between environmental risk perception and cultural worldviews was negative except for egalitarianism, and the correlation between hierarchy and environmental risk perception was higher than the others, while individualism was higher than fatalism. Heightened environmental risk perception mediates the relationship between egalitarianism and waste sorting. Reduced environmental risk perception mediates the relationship between hierarchy and waste sorting, and mediates the relationship between individualism and waste sorting.

**Conclusion:**

These new findings provide initial support for the mediating role of environmental risk perception in the relationship between cultural worldviews and waste sorting. Both theoretical and practical implications for understanding the psychological mechanisms of waste sorting are discussed.

## Introduction

As the global population grows and urbanization accelerates, the generation of municipal solid waste is increasing at an unprecedented rate. A study published by the World Bank estimates that the annual generation of municipal solid waste will reach 340 million tons worldwide by 2050 (1). This challenge not only puts enormous pressure on the environment but also poses a serious test for public health and resource recycling. Waste sorting, as an effective waste management strategy, has become a key measure to address this issue in several countries and regions around the world (2).

Japanese scholars first started to study the waste-sorting behavior of residents in the 1960s (3). Subsequently, the United States, Germany, and other countries also began to research the factors affecting urban dwellers’ waste-sorting behavior (4, 5). In 1992, the concept of waste sorting was first introduced with the “Notice on several opinions on solving the problem of urban waste in China,” which was issued by the Ministry of Construction of China along with other three departments. The first leading cities to implement waste sorting, eight in total, including Beijing and Shanghai, have studied waste sorting management methods, and have gained some practical experience. However, there was a lack of public awareness regarding waste sorting, and the majority of the cities’ sorting work was formalistic, making it difficult to establish a systematic and long-term management mechanism. Thus, waste reduction has been implemented at the source to alleviate the challenge of garbage encircling cities.

In China, municipal waste is still mainly disposed of in landfills and incinerated (6). Landfills occupy a large area and have a limited lifespan, which can easily result in conflicts between urban dwellers and landowners, while waste incineration is prone to producing exhaust gasses, such as particulate matter, sulfur dioxide, and sulfur trioxide, which can cause air pollution (7). Hence, the “Implementation Plan for the Domestic Waste Sorting System,” was issued in March 2017 by the China National Development and Reform Commission and the Ministry of Housing and Urban–Rural Development, making it mandatory for residents to separate domestic waste for the first time. In addition, the Ministry of Construction also issued the “Implementation Plan for the Household Waste Sorting System,” making it mandatory for residents to sort their household waste for the first time (8). In fact, in addition to being driven by macro factors, such as government policies and infrastructure (9), residents’ engagement in household waste sorting is also influenced by individual-level variables (10). Risk perception is identified by risk perception theory as an important factor influencing individual behavior (11). The Value-Belief-Norm (VBN) theory suggests that personal values are important factors in influencing pro-environmental behavior intentions (12). However, few studies have tested the cultural theory of risk in the Asian context including China. Compared to Western countries, Chinese culture, politics, economy, and community social support differ significantly, so there is a need to determine whether the standard account of cultural theory is applicable in Asia. Therefore, this study attempts to examine the influence of two individual-level variables, cultural worldview, and environmental risk perception, on urban Chinese waste sorting and their mechanisms of action from the VBN theory and risk perception theory, and further proposes the optimization of urban dwellers’ waste sorting.

### Theory and hypotheses

#### Cultural worldviews and waste sorting

Cultural Theory (CT), also known as Grid-Group Cultural Theory (GGCT), is a widely used framework for analyzing culture (13). It was developed by Durkheim and expanded by Douglas, Wildavsky, and others (14–21). CT uses two dimensions - Grid and Group - to represent a culture’s reliance on standardized rules and the integration of individual interests into group interests (22). These dimensions can be positive or negative, creating four quadrants: Hierarchy (grid+ and group+), individualism (grid- and group–), egalitarianism (grid– and group+), and fatalism (grid+ and group–) (23). Each quadrant represents a different approach to addressing social challenges and risks. Hierarchy emphasizes rules and order within a group (24). Egalitarianism focuses on equality and social justice (25, 26). Individualism values self-regulation over authority (24, 27). Fatalism believes that challenges are beyond one’s control (28).

Waste sorting refers to the process of recycling and treating garbage according to its different components, attributes, utility value, environmental impact, and the requirements of different treatment processes (29). From the early days of simple sorting to today’s sophisticated separation, the concept and practice of waste sorting have continuously developed and improved. Many countries and regions have formulated relevant laws and regulations to promote the popularization and implementation of waste sorting (30). Waste sorting helps reduce pollution and promotes the balance and restoration of the ecosystem (31). Waste sorting promotes the development of a circular economy, improves resource utilization efficiency, and reduces production costs (32). In addition, waste sorting promotes the development and innovation of related industries, providing a new impetus for economic growth (33). In terms of culture, waste sorting has raised public awareness of environmental protection (34), promoted the popularization of green lifestyles, and helped build a social and cultural atmosphere in which human beings live in harmony with nature.

The specific presentation of cultural worldviews in social life forms a cultural way of life. This cultural lifestyle reflects individual cultural worldviews (35). Mary Douglas’s cultural symbolic analysis of pollution and taboos points out that when something is considered unclean, it is due to the misplacement of people’s cognitive classification, which is related to the construction of social order (36). This view inspired the later research on garbage, that is, attention to the cultural connotation and value of garbage. Thompson proposed the Rubbish Theory (RT). According to Thompson, our worldview determines our actions. Wang et al. (37) proposed that people develop behavioral habits that correspond to their cultural worldview. Each of the four cultural worldviews will influence decision-making and behavior including waste sorting (38). Zeng et al. showed that egalitarianism was significantly positively correlated with pro-environmental behavior, and individualism was not significantly correlated with pro-environmental behavior (39). Toorzani and Rassafi showed that egalitarianism, hierarchy, and pro-environmental behavior were significantly positively correlated, individualism was significantly negatively correlated with pro-environmental purchasing behavior, and fatalism was not significantly correlated with pro-environmental purchasing behavior (40). Jung and Cho showed that individualism and pro-environmental purchasing behavior were significantly positively correlated (41). Waste sorting, as a type of pro-environmental behavior, has certain commonalities with other pro-environmental behaviors in terms of the mechanism of influence (42, 43). Based on the above theories and the relationships between the variables, the following hypotheses were formulated in this study.

Hypothesis 1a: Higher egalitarianism and hierarchy are related to higher waste sorting.

Hypothesis 1b: Higher individualism is related to lower waste sorting.

Hypothesis 1c: Fatalism is non-significantly related to waste sorting.

#### Environmental risk perception and waste sorting

Beck introduced the concept of risk society in 1980. He argued that every period of human history has been a risk society, facing different threats at different times, and that in modern society, humans have gradually become the main creators of risk (44). The rapid development of technology has solved many challenges that were previously considered high-risk by humans and has made life easier. However, it has also resulted in numerous environmental hazards, disasters, and social inequalities, in addition to a significant number of unpredictable but far-reaching unknown risks (45, 46). Blaylock further noted that the more uncertainty an individual perceives, the greater the perceived risk (47). Maartensson and Loi’s empirical study of Australian adults found a significant positive correlation between environmental risk perception and pro-environmental behaviors (48). Han et al. also found a significant positive correlation between environmental risk perception and pro-environmental behavior in a survey study of Chinese adults (49). The more aware individuals are of environmental risks, the more likely they are to engage in environmentally-friendly behavior (39). When individuals perceive environmental risks in their surroundings, they may tend to modify their behavior in order to promote environmental well-being. Furthermore, waste sorting, as a form of pro-environmental behavior (42, 43), may be influenced by the perception of environmental risks. Thus, this study proposes the following hypothesis.

Hypothesis 2: An individual who perceives environmental risk more strongly has a greater tendency to sort waste.

#### Cultural worldview, environmental risk perception, and waste sorting

Attitudes toward risk differ among individuals who hold different values (50). Research has shown that egalitarianism is positively associated with environmental risk perception and policy support (24, 51), while individualism and hierarchy are negatively associated with these factors (52–54). A number of studies showed that fatalism is negatively correlated with risk perception (55). Fatalism and environmental risk perception are not significantly correlated in most studies (21).

The VBN theory, proposed by Stern et al., suggests that environmental attitude variables are influenced by an individual’s value system (56). The theory proposes that individual values influence beliefs, which in turn result in different behaviors. Beliefs are seen as an important link between values and behaviors. Therefore, the influence of cultural worldview on behavior may not be entirely direct (57) but it may instead affect people’s behavior through changes in their perceptions (39, 58). Lacroix and Gifford’s Canadian study found that climate change risk perceptions play a mediating role between cultural worldviews and barrier perceptions (59). The study by Zeng et al. revealed that risk perception plays a mediating role between cultural worldview and pro-environmental behavior (39). Based on the existing relationship between the four dimensions of cultural worldview and environmental risk perception, waste sorting, this study proposes the following hypotheses.

Hypothesis 3a: The relationship between egalitarianism and waste sorting is mediated by an increased environmental risk perception.

Hypothesis 3b: The relationship between hierarchy and waste sorting is mediated by a reduced environmental risk perception.

Hypothesis 3c: The relationship between individualism and waste sorting is mediated by a reduced environmental risk perception.

## Methods

### Participants and procedures

A convenience sampling method was used to conduct the survey. Data were collected online from 15 March 2021, to 25 March 2021, using the Wenjuanxing, a widely accepted online questionnaire survey platform in China. In nine provinces and cities, 783 questionnaires were distributed to urban dwellers, including Sichuan, Chongqing, Yunnan, Guangdong, Hainan, Liaoning, Shandong, Shaanxi, and Inner Mongolia. Four undergraduate students majoring in psychology were trained as examiners to be familiar with the issues to be addressed in the questionnaire, which was administered both in batches and collectively. After the survey, the questionnaires were collected on the spot. The survey was rigorously designed and conducted in adherence with national and international ethical guidelines including ethical approval, informed consent, and data integrity. In addition, our study strictly respected the participants and protected their privacy and confidentiality. This research was approved by the Research Committee of Sichuan Institute of Industrial Technology (SCGKY-002) in accordance with international ethical standards.

The average response time was 8 min and 23 s, with questionnaires with a response time of fewer than 3 min, regular responses (e.g., always as a pattern of 1, 2, 3, 4, 5), and straight liners deleted. Thus, 744 questionnaires were valid, with an effective rate of 95.02%. There were 371 males (49.87%) and 373 females (50.13%). The ages of the participants ranged from 18 to 70 years (mean age 35.45 years, SD 15.91 years). The average monthly household income was less than RMB 2,000 for 233 people (31.32%), RMB 2,000–4,000 for 311 people (41.80%), RMB 4,001–6,000 for 116 people (15.59%), RMB 6,001–8,000 for 39 people (5.24%), RMB 6,001–8,000 for 23 people (3.09%) and more than RMB 10,000 for 22 people (2.96%).

### Measures

#### Waste sorting behavior scale

Waste sorting was measured using the Waste Sorting Behavior Scale developed by Han et al. (60) which consists of four items. This scale measures residents’ waste sorting behavior in relation to food waste, recyclable waste, and other waste sorting behaviors. Higher scores indicate higher waste sorting behavior according to a 5-point Likert scale. The Cronbach’s coefficient here was 0.81. The goodness of fit indices for this scale showed a reasonably good fit, *χ2/df* = 2.63, CFI = 0.995, TLI = 0.974, and RMSEA = 0.06.

#### Cultural worldview scale

Scholars of cultural theory debate whether individual cultural biases should be measured by four separate indices or classified into four quadrants (61). This paper argues for treating individuals as hybrids of the four cultural types and proposes the use of separate indices to measure each culture (62). Scholars have also suggested that combining measures of worldview and relational statements provides better validity for measuring culture (63). The Cultural Worldview Scale developed by Zeng et al. (39) was used in this study to measure cultural worldview. The scale has four dimensions: fatalism, individualism, hierarchy, and egalitarianism. It consists of four items that measure the following dimensions: (1) no motivation and irrelevant, (2) self-interest, (3) laws and rules, and (4) respect for nature as a human being, measuring fatalism, individualism, hierarchy, and egalitarianism. Item number 4 is a ranking question: “Sequencing responsibility for environmental protection in Government/Business Corporations/Everyone/Other“with 5, 4, 3, and 2 points for the first to fourth place, and 1 point for the unranked option. The Cronbach’s α coefficient for the egalitarianism, hierarchy, individualism, and fatalism indices was found to be 0.23, 0.11, 0.10, and 0.30, respectively. The goodness of fit indices for this scale showed an acceptable fit: *χ2/df* = 3.28, CFI = 0.913, TLI = 0.926, and RMSEA = 0.07. Despite the plausible validity of our cultural measures, the Cronbach’s coefficient for each of the four cultural worldviews is very low, which may lead to unreliable measurement (64).

#### Environmental risk perception scale

The Environmental Risk Perception Scale developed by Wang (65) was used in this study to measure environmental risk perception. It consists of six items, such as “How serious is the problem of domestic waste pollution in your area?” Higher scores indicate a higher perception of environmental risk. The Cronbach’s coefficient here was 0.89. The goodness of fit indices for this scale showed a reasonably good fit, *χ2/df* = 2.78, CFI = 0.991, TLI = 0.963, and RMSEA = 0.06.

### Statistical analysis

The data were statistically analyzed using SPSS 24.0 for descriptive statistics, correlation analysis, and tests of variance. Additionally, MPLUS 7.0 was used for structural equation modeling to examine the role of environmental risk perception in the relationship between fatalism, individualism, hierarchy, egalitarianism, and waste sorting.

## Results

### Common method deviation test

When data are collected using the self-report method, the issue of common method bias may arise. Following the recommendations of Zhou and Long (66), we implemented relevant controls in the test. These controls included using reverse presentation for some items and emphasizing that there is no right or wrong answer. Additionally, a common method bias test was conducted using the Harman one-way test prior to analyzing the data. The results showed that there were nine factors with eigenvalues greater than one, explaining 66.14% of the variance. The first factor explained 18.48% of the variance, which is below the 40% threshold (67). There is, therefore, no significant common method bias in this study.

### Analysis of correlations

An overview of all variables is presented in Table 1 along with their zero-order correlations. Waste sorting was found to be relatively insignificantly negatively correlated with fatalism and individualism. All cultural worldviews, except egalitarianism, were negatively correlated with environmental risk perception. Hierarchy was more highly correlated than others, while individualism was more highly correlated than fatalism. Waste sorting was found to be significantly influenced by gender, while fatalism was significantly negatively associated with gender. Therefore, we chose “gender” as a control variable in the structural equation modeling.

**Table 1:** Descriptive statistics and correlations between waste sorting, environmental risk perception, and cultural worldview.

Variables	*M*	SD	1	2	3	4	5	6	7
1. Gender^a^	–	–	1						
2. Waste sorting	4.549	0.874	0.103**	1					
3. Environmental risk perception	2.187	0.708	−0.068	0.286***	1				
4. Fatalism	0.679	0.372	−0.076*	−0.020	−0.037	1			
5. Individualism	1.370	0.219	−0.061	−0.007	−0.181***	0.122**	1		
6. Hierarchy	1.522	0.272	0.014	0.100**	−0.197***	0.152***	0.096**	1	
7. Egalitarianism	2.201	0.271	0.006	0.136***	0.317***	−0.141***	−0.111**	−0.364***	1

^a^Gender is a dummy variable, women = 1, men = 2; *p < 0.05, **p < 0.01, ***p < 0.001.

### Structural results

**Figure 1:**
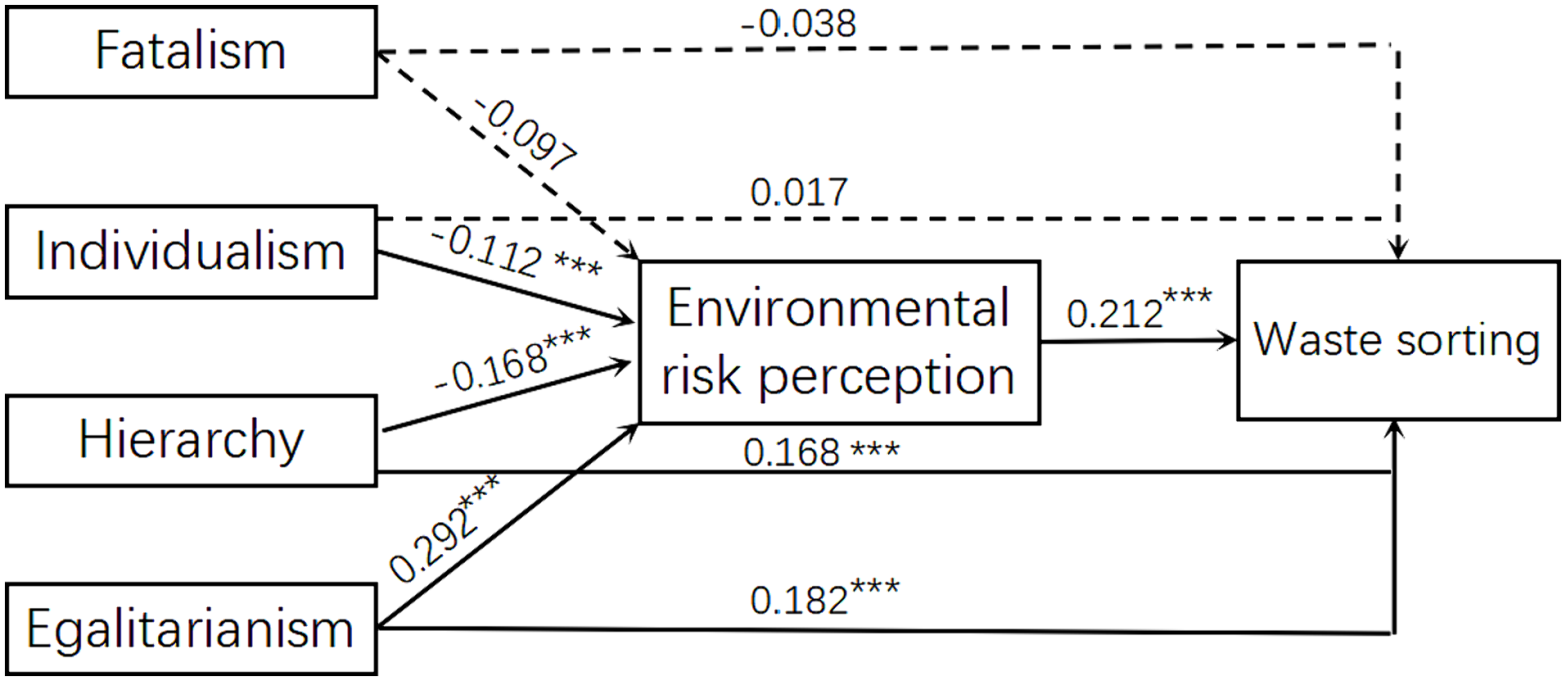
Path analysis of the model.

Structural equation modeling (SEM) was used to test the research hypotheses and the model estimation method was maximum likelihood estimation. Structural equation analysis was conducted using fatalism, individualism, hierarchy, and egalitarianism as predictor variables, waste sorting as the outcome variable, environmental risk perception as the mediating variable, and gender as the control variable. Significance tests were conducted using the Bootstrap method of bias correction, with 5,000 replicate samples with replacement, to determine whether the mediating effect was statistically significant. The test was based on whether the 95% confidence interval included 0. The mediating effect was significant if the confidence interval did not include 0, and not significant if it did. The test results of the hypothesis model showed good overall model fit indices: *χ*^2^ = 11.652, *df* = 4, RMSEA = 0.074 [90% CI: 0.063, 0.085], CFI = 0.932, TLI = 0.926, SRMR = 0.039. The specific path coefficients are shown in Figure 1.

### Mediating effect analysis

The model was further tested using the Bootstrap method of bias correction. The results (Table 2) indicate that all mediating effects are non-zero, except for the 95% confidence interval for the mediating effect of fatalism→environmental risk perception→ waste sorting, which includes 0, indicating a significant mediating effect. In other words, the effects of environmental risk perception, individualism, hierarchy, egalitarianism, and garbage classification were mediated by values of −0.024, −0.036, and 0.063, respectively. Hypotheses 3a, 3b, and 3c are therefore confirmed.

**Table 2:** Bootstrap results for each path coefficient of the hypothetical model.

Effect	Path relationships	95% confidence interval	Intermediary effect value
Direct effect	Hierarchy→waste sorting	[0.091, 0.245]	0.168
	Egalitarianism→waste sorting	[0.097, 0.267]	0.182
Intermediary effect	Individualism→environmental risk perception→waste sorting	[−0.034, −0.014]	−0.024
	Hierarchy→environmental risk perception→waste sorting	[−0.046, 0.026]	−0.036
	Egalitarianism→environmental risk perception→waste sorting	[0.055, 0.071]	0.063

## Discussion

### The relationship between egalitarianism, hierarchy and waste sorting

The findings suggest that egalitarianism and hierarchy are positively related to waste sorting, which is consistent with the hypothesis of 1a. Egalitarianism sees the object as equal to others, views nature as fragile and resources as limited, and is more willing to participate in environmental protection and waste sorting. Hierarchy honors specialists and authority and is more acceptable to environmental specialists or government departments (68). As local governments have been increasing their efforts to publicize environmental risks in recent years (69), it is more conducive for hierarchies to engage in positive behavior in waste sorting.

### The relationship between individualism and waste sorting

Individualism is non-significantly negatively related to waste sorting, which is inconsistent with the hypothesis of 1b. Individuals who hold individualistic cultural worldviews perceive environmental risks as symbolic representations of social elite status and authority. They argue that an excessive fear of environmental risks is a form of criticism of social elites, which in turn poses a threat to their cultural worldviews (37). They are more likely to devalue environmental protection and less likely to engage in pro-environmental behavior.

### The relationship between fatalism and waste sorting

Fatalism is non-significantly related to waste sorting, which is consistent with hypothesis 1c. This is consistent with Dake’s study (28), which found that fatalists tend to believe that social problems are largely beyond their control. As a result, fatalists typically do not participate in individual waste sorting.

### The relationship between environmental risk perception and waste sorting

Environmental risk perception is positively related to waste sorting, which supports hypothesis 2. This is in agreement with previous results (70). That is, the stronger the public’s perception of environmental risk, the more likely people are to engage in environmental protection activities and to participate more frequently in waste sorting (71). Individuals with a higher perception of environmental risk exhibit more pro-environmental behavior compared to those with a lower perception of environmental risk. And when people perceive environmental risks around them, they may be more likely to adjust their behavior to benefit the environment.

### The mediating role of environmental risk perception

In this study, SEM was used to investigate how environmental risk perception mediates the relationship between waste sorting and the four cultural worldviews. In addition to directly influencing waste sorting, egalitarianism can also increase waste sorting by enhancing environmental risk perception, indicating that risk perception plays a significant role. This supports hypothesis 3a. CT suggests that individuals assess and respond to risk differently depending on their preferred social organizational structure or cultural worldviews (72). Egalitarianism may show greater fear of human-generated harm (reflected in larger positive effect sizes) than natural disasters because it provides a stronger basis for arguing for greater regulation and social reform to reduce social disparity (52). Not only is hierarchy directly related to waste sorting, but it can also increase waste sorting behavior by reducing environmental risk perceptions, suggesting that risk perceptions play a partial mediating role in this process. This supports hypothesis 3b. According to Xue et al. (53), hierarchies function to maintain power structures and disparities within society. Therefore, they may fail to recognize the dangers posed by this system. Even in the presence of high risk, hierarchical behavior is not significantly affected. The negative association between individualism and waste sorting is relatively insignificant but may increase waste sorting behavior by reducing environmental risk perceptions, suggesting that risk perceptions play a partial mediating role in this process. This supports hypothesis 3c. The preference for less regulation and more freedom may stem from the fact that individuals are less concerned about human-generated hazards than natural hazards (53). Individualistic perceptions of environmental risk are inhibited, leading to a decrease in waste-sorting behavior.

### Limitations and future research

While this study contributes to our understanding of cultural worldviews in China, it has some limitations that should be acknowledged. First, urban dwellers in nine provinces and cities were sampled using convenience sampling, which may limit the generalizability of the results. Future research should consider expanding the sample to include both rural and urban dwellers from different regions of China. Second, this study relied primarily on self-reported measures, which may have introduced biases such as social desirability and recall bias. Future research should consider using alternative methods such as experience sampling and peer evaluation to supplement self-report measures. Third, Cronbach’s α coefficients for each of the four cultural worldviews were low, indicating unsatisfactory reliability. Future research can improve the validity and reliability of the study by conducting tests and making revisions to the items measuring the four cultural worldviews (64). Fourth, the dimensions of cultural worldviews may not be independent of each other, which is consistent with previous research in cultural theory. The results of our study should be replicated in future studies using other worldview scales, such as those developed by Kahan et al. (73). Additionally, it is important to consider that the worldview measures used in this study were developed in the United States and may not accurately reflect Chinese public opinion. Future studies should examine cross-cultural measurement invariance or develop new culturally appropriate measures.

## Conclusion

Using RT and VBN theory, we have developed a theoretical framework that establishes a connection between cultural worldviews, environmental risk perception, and waste-sorting behavior among urban dwellers. The results showed that an increased perception of environmental risk contributes to the improvement of waste sorting practices in terms of egalitarianism. Reduced environmental risk perception is conducive to improving waste sorting practices and promoting individualism. We gained a deeper understanding of how urban dwellers sort their waste in this study. It also provides empirical evidence for the city manager to effectively intervene in waste sorting among urban dwellers.

## Data availability statement

The original contributions presented in the study are included in the article/supplementary material, further inquiries can be directed to the corresponding author.

## Ethics statement

The studies involving humans were approved by this research was approved by the Research Committee of Sichuan Institute of Industrial Technology. The studies were conducted in accordance with the local legislation and institutional requirements. The participants provided their written informed consent to participate in this study.

## Author contributions

LC: Writing – original draft, Writing – review & editing. QL: Writing – original draft. EW: Methodology, Writing – review & editing. ML: Conceptualization, Writing – review & editing. ST: Writing – review & editing, Formal analysis, Validation.
